# Using substantial reductant concentration with chelation therapy to enhance small aggregate dispersal, iron mobilization, and its clearance in neurodegenerative diseases

**DOI:** 10.3389/fnins.2022.1006203

**Published:** 2022-09-15

**Authors:** Barry B. Muhoberac

**Affiliations:** Department of Chemistry and Chemical Biology, Indiana University-Purdue University Indianapolis, Indianapolis, IN, United States

**Keywords:** neurodegeneration, iron chelation-reductant therapy, aggregate dispersal, ascorbate, glutathione, ROS

## Abstract

Connections between altered iron homeostasis and certain neurodegenerative diseases are highlighted by numerous studies suggesting iron neurotoxicity. Iron causes aggregation in neurodegenerative disease-linked proteins as well as others and additionally facilitates oxidative damage. Iron and oxidative damage can cause cell death including by ferroptosis. As treatment for neurodegeneration, chelation therapy alone is sometimes used with modest, varying efficacy and has not in general proven to reverse or halt the damage long term. Questions often focus on optimal chelator partitioning and fine-tuning binding strength; however iron oxidation state chemistry implies a different approach. More specifically, my perspective is that applying a redox-based component to iron mobilization and handling is crucial because ferrous iron is in general a more soluble, weaker biological binder than ferric. Once cellular iron becomes oxidized to ferric, it binds tenaciously, exchanges ligands more slowly, and enhances protein aggregation, which importantly can be reversed by iron reduction. This situation escalates with age as brain reducing ability decreases, iron concentration increases, autophagic clearance decreases, and cell stress diminishes iron handling capacity. Taken together, treatment employing chelation therapy together with a strong biological reductant may effectively remove inappropriately bound cellular iron or at least inhibit accumulation. This approach would likely require high concentration ascorbate or glutathione by IV along with chelation to enhance iron mobilization and elimination, thus reducing cumulative cellular damage and perhaps restoring partial function. Potential treatment-induced oxidative damage may be attenuated by high reductant concentration, appropriate choice of chelator, and/or treatment sequence. Comprehensive study is urged.

## Introduction

Elevated iron levels are associated with several neurodegenerative diseases through numerous *in vivo* and *in vitro* studies (Singh et al., 2014). Excess iron deposition has been mapped to specific regions in the brain associated with particular neurodegenerative disease characteristics through histology and magnetic resonance imaging. Cultured cells behave abnormally when exposed to excess iron as do organelles. Even the most basic system of purified neurodegeneration-related proteins behaves in a toxic manner. For example, Alpha-synuclein, which is the main component of Lewy bodies in Parkinson’s disease not only binds iron, but aggregates under iron exposure. The key proteins implicated in Alzheimer’s disease, Tau and A-Beta, also bind iron causing aggregation and fibrilization. This kind of inappropriate iron binding directly induces aggregation and does not require the addition of reductants or other cellular components. Another kind of inappropriate iron binding becomes the catalytic center for generation and interconversion of various reactive oxygen species (ROS) causing cellular oxidative damage, which may be an additional important, but more indirect contributor to protein aggregation. If aggregates are not cleared, deposits are formed, and these are visible microscopically in several neurodegenerative diseases. *It is most likely that soluble aggregates and perhaps the surface of deposits contain exposed, inappropriately bound iron (IBI) that fosters further aggregation, additional iron binding, and enhanced ROS formation in a cyclical manner.* It is up to cellular processes at several levels to prevent this aberrant behavior, but in doing so, this taxes the cells both energetically and materially (Muhoberac and Vidal, 2019). The ability of the autophagy-lysosome system to remove aggregates declines with age (Karabiyik et al., 2021). Cell death by ferroptosis is an iron-dependent process, implicated in both neurodegeneration and aging, that is marked by ROS-centered phospholipid damage and stopped by various compounds including iron chelators (Vitalakumar et al., 2021).

Many cellular studies show aberrant molecular level behavior caused by excess iron. *Thus, it would be curious if diverse neurodegenerative diseases all result in iron elevation, aggregate formation, and deposition if iron was not causally involved in some stage of the disease in some cases.* Thus, a logical approach to a potential treatment for neurodegeneration is to carefully remove toxic excess iron in a manner not jeopardizing normal iron-dependent pathways, shifting aggregation damage elsewhere, or producing excessive ROS. This approach has been attempted through chelation therapy for decades and has provided in some instances partial success, but not a robust treatment to reverse or halt neurodegenerative damage long term. *This lack of success suggests that some key aspect of the approach is being overlooked* prompting my perspective on what can be chemically deduced, studied experimentally, and then potentially applied clinically to move the situation forward. Although I am writing this perspective as a general approach to iron involvement in neurodegeneration, the diverse biochemistry of neurodegenerative diseases suggests that the approach may be substantially more effective in one or more specific ones than others. Furthermore, considering the gradual accumulation of iron aggregates and the potentially protective nature of macroscopic deposits, attempts at abrupt removal may be problematic and thus require more long-term, measured clinical treatment.

## Ferric and ferrous iron binding characteristics

The benefit physiologically of iron as a major biochemical cofactor is its diversity in ligand binding and reactivity. However, the same diversity can occasionally become problematic fostering unwanted iron-ligand binding and reactions in its routine transport, utilization, and elimination. Iron switches between two oxidation states relatively easily with cellular reductants and available oxygen, and their chemistry differs widely. Both redox states, ferric (Fe^3+^) and ferrous (Fe^2+^), can themselves have different electron configurations, high- and low-spin, which depend on the ligands bound to the iron. Ferrous iron is relatively soluble with bound water exchanging rapidly, but not ferric, which is basically insoluble at neutral pH and fosters iron hydrolysis, aggregation, and precipitation (Crichton, 2009) in addition to protein aggregation. The cytosol is believed under normal conditions to provide sufficient reductants and endogenous small binding molecules to keep iron soluble and prevent IBI attachment, but this may deteriorate with age. As a complication, IBI if ferrous can generate ROS and cause oxidative damage. These solubility and reactivity problems explain why cellular iron carriers, either proteins (e.g., transferrin) or small cytosolic molecules, and the storage protein ferritin are needed for safe handling. Such *appropriately bound iron is effectively protected from participating in (1) direct, iron binding-induced protein aggregation (e.g., forming iron bridges between proteins)*, *and (2) more indirect, secondary aggregation from iron binding-induced ROS production and resulting protein damage, as will be discussed*.

Understanding the potential and preference for ligand binding to ferric or ferrous iron is clearly important. Chemists have classified metal ions and ligands as hard or soft, which predicts bond formation favorability, or crudely, the strength of the bond for metal-ligand pairs (Bertini et al., 2007). Major influencing factors are metal oxidation state and the ligand’s chemical group directly binding the iron. Ferric iron prefers to bind to oxygen atoms as found in the carboxylate and phenolic groups, whether they are connected to small cytosolic molecules or part of the amino acids aspartate, glutamate and tyrosine in proteins. Ferric iron also preferentially binds the oxygen in alcohols, and certain amine nitrogen and phosphate groups. Ferrous iron binds to oxygen atoms in ligands but not with the strength of ferric. Ligands containing sulfur (e.g., cysteine, methionine, and other mercaptans) and some ring nitrogens (e.g., histidine and the chelator 1,10-phenanthrolene) are favored by ferrous over ferric binding, but generally not greatly. The larger number of amino acids found in average globular proteins with groups that prefer to bind to ferric iron makes it problematic because of aggregation induction, as will be discussed. Additionally, brain reductant levels decrease with age (Lykkesfeldt and Moos, 2005), again enhancing the likelihood of ferric iron binding. The difference in binding strength from oxidation state can be substantial where, for example, in Alpha-synuclein the ferric iron binding constant is 10^+13^ M^–1^ and ferrous is only 10^+4^ M^–1^ (Peng et al., 2010).

## ROS generation

Iron is a well-known catalytic center for generation and transformation in form of ROS (e.g., peroxide and hydroxyl radical) in biological systems. There generation can originate from the misfiring of normal catalytic reactions to which cells have adapted and synthesized ROS disposal proteins for their handling under routine circumstances (Muhoberac, 2020). However, ROS can be generated *de novo* cellularly from IBI that is improperly coordinated and exposed to molecular oxygen and reductant. The concept of “proper coordination” stems from the preferred octahedral geometry and hexacoordination of ferric and ferrous iron, with all 6 ligand group attachment sites occupied by protein or carrier ligands shielding the iron from unwanted chemical reactions (Graf et al., 1984). If one or more of the sites is open to molecular oxygen binding, ROS generation is facilitated. Such aberrant ROS generation is usually described in part by Fenton or Haber Weiss reactions. These processes generate ROS cyclically with a single improperly coordinated iron because cells are rich in reductant and oxygen to feed the reaction, multiplying potential cellular damage substantially. The concentration of reductants in the brain is normally high (millimolar ascorbate), but free iron is not. Additionally, depending on identity, ROS can cause either nearby protein damage or diffuse substantial distances causing non-local protein or phospholipid damage. Modified phospholipids can lead to ferroptosis. Damaged proteins can become destabilized and unfolded producing additional IBI binding sites enhancing both direct iron binding-induced protein aggregation and ROS generation from additional improperly coordinated iron. Chelators in general can remove iron and provide proper coordination. It should be noted that some enzymes become 5-coordinate as part of their normal catalytic processes, but here the identity of what binds to the iron is highly controlled by the iron ligands and binding cavity geometry imposed by protein structure.

## Clinical iron chelators

Chelators have more than one metal ion binding group synthesized into a single molecule, making them bidentate, tridentate, etc. binders, thus increasing their binding strength through entropic effects. Three common iron chelators in clinical use are deferiprone (Ferriprox, DFP), deferasirox (Exjade, DFX), and desferrioxamine (Desferal, DFO), each with different chemical binding and physiological properties such as absorption, half-life, and lipid partitioning (Crichton, 2009). DFP is a small, single ring compound that provides two oxygen groups for iron binding and is a bidentate chelator. Thus for hexacoordinate (i.e., properly coordinated) iron, three DFP molecules are required (DFP:Fe of 3:1). Importantly, if the concentration of available chelator is low, e.g., during plasma clearance, molecular oxygen binding sites on iron could open for ROS generation and/or unwanted binding to proteins. DFP bound at a 1:1 or 2:1 DFP:Fe ratio can produce ROS, which is attenuated at 3:1 (Devanur et al., 2008) highlighting the importance of chelator initial concentration and half-life. DFX is tridentate and binds in a DFX:Fe of 2:1 for hexacoordination. DFO is a linear chain that wraps around chelating iron in all 6 coordination sites (DFO:Fe of 1:1). Importantly, it is not just binding strength of the chelator but also its concentration that dictates its ability to remove iron to reverse aggregation and block ROS formation. Interestingly, ethylenediaminetetraacetic acid (EDTA), a well-known chelator, can enhance ROS generation apparently because it becomes 7-coordinate through distortion and binds molecular oxygen (Mizuta et al., 1993).

## Iron protein binding, aggregation, and reductant-induced resolubilization

It is ferric iron that is strongly implicated in the aggregation and deposition of key neurodegenerative disease-related proteins, as well as proteins in general. Ferric iron binds to A-Beta through the phenolic oxygen of tyrosine in addition to carboxylate groups of aspartate and glutamate, and causes aggregation (Miura et al., 2001). Ferric iron, but not copper causes this, and chelation can reverse this aggregation (Tahmasebinia and Emadi, 2017). Protein oligomer formation is induced in Tau by ferric iron, but not by a series of divalent transition metal ions (Bader et al., 2011). Ferric iron binds to Alpha-synuclein tightly unlike ferrous and aggregates it into a sodium dodecyl sulfate-resistant form whereas ferrous iron does not. These aggregations of purified proteins are matched with brain deposits in animal and human studies. Interestingly, a model of thrombosis was developed using ferric chloride treatment, which enhances aggregation of plasma components and red blood cells, that is dependent only on chemical charge-based interactions, not thrombolytic cascade (Ciciliano et al., 2015). In industry, addition of ferric chloride is used for bulk protein precipitation and recovery. For example, proteins are precipitated from fruit extracts with ferric chloride addition (Bártová and Bárta, 2009). This direct, binding-induced aggregation requires only ferric iron without the complication of reductant addition producing ROS.

Ferric iron-induced protein precipitation can be reversed with reductant addition. For example, the protease caldolyn can be precipitated from a protein solution by addition of ferric iron, and then resolubilized by reducing the iron with dithionite and addition of the chelator citrate (Collingwood et al., 1988). Hyperphosphorylated Tau is precipitated by addition of ferric iron but not ferrous, and the aggregation is reversed by reducing the iron (Yamamoto et al., 2004). Even the mineral core of the iron storage protein ferritin is redissolved into solution by the reductant ascorbate (Bienfait and Van Den Briel, 1980). *Clearly, both the presence and oxidation state of iron is crucial to the stability of cellular aggregates, with ferric iron reduction able to disaggregate and disperse them*.

## Protein aggregation drivers and mechanisms

Statistically, ferric iron has a larger likelihood of protein binding because (1) average protein composition has a greater number of amino acids that prefer ferric iron binding versus ferrous and (2) they generally bind more tightly. Globular proteins have higher aspartate, glutamate, and serine percentages (versus histidine and sulfur-containing amino acids) substantially favoring protein ligand group oxygen availability, thus enhancing probability of ferric iron to protein interactions and binding. Additionally, ferric iron hydrolysis creates ferric iron hydroxide complexes that can hydrogen bond and bridge with the abundant, linear nitrogen-containing amino acids. This situation is made more critical because with age brain reductant levels decrease and iron increases, again favoring ferric iron binding. *These drivers favor direct, ferric iron binding-induced aggregation* of *transiently unfolded native or permanently unfolded (denatured) proteins.*

The simplest aggregation mechanism would be iron bridging, where amino acids (e.g., carboxylates) on separate proteins become bridged by one or more IBIs caused by high concentrations, unusual proximity, or transient unfolding (Muhoberac and Vidal, 2013). This bridging itself could shift protein unfolding equilibrium creating additional iron binding sites and interactions with more proteins. Thus, aggregation could produce additional iron binding sites that cause more aggregation, and as a secondary process through improper iron coordination, produce ROS. Protein oxidative damage changes the structure and charge of amino acids potentially becoming destabilizing enough to permanently unfold proteins, further enhancing iron-centered aggregation and ROS generation, cyclically. Additionally, unfolding may cause hydrophobic association between exposed interior amino acids contributing to aggregation by a different interaction making disaggregation more difficult once it is established. *Although ROS-centered damage is often the research focus in neurodegenerative diseases, excess and IBI causing aggregation is likely the primary pathological driver, and if eliminated, ROS formation would also be reduced.*

## Reduction, iron mobilization, chelation, and elimination

Results from using ascorbate alone in treatment of diseases, including neurodegeneration, are equivocal and often contradictory, and like chelation therapy have not led to a clear decision on efficacy. This likely is from (1) low administration dosage, (2) the rapid ascorbate half-life (2-3 hours), and (3) plasma concentration that is self-limiting from oral administration, which is substantially less than that achievable by intravenous treatment (IV). Considering the molecular level studies outlining less likely and weaker binding of ferrous iron to proteins and resolubilization of ferric iron binding-induced precipitated proteins by reductant addition, *it can be deduced that with neurodegeneration, combining a strong biological reductant with chelation therapy should disperse iron-protein aggregates not dispersible by chelation therapy alone and facilitate iron elimination.*

Next, upon literature examination I found substantial support for the feasibility of this approach from clinical, non-neuronal iron overload studies where the efficacy of iron elimination is enhanced substantially by simultaneous administration of chelator and ascorbate. In a glucose deficient mutant rat model with iron overload established by iron-dextrose injections, oral ascorbate enhanced hepatic iron removal by DFX 21% (Brewer et al., 2012). With a carbonyl-iron, dietary supplementation-induced, overload rat model of cardiotoxicity examining DFO (subcutaneously) and DFP (orally), a significant reduction in serum iron levels and markers of oxidative stress over those achieved by chelators alone occurred with oral ascorbate (Emara et al., 2006). In a urinary iron excretion study in patients receiving regular blood transfusions, iron removal by subcutaneous DFO treatment was increased by 24–245% (mean 96%) with an oral 2-gram dose of ascorbate (Hussain et al., 1977). IV DFO-induced urinary iron excretion in the iron overload disease hemochromatosis is increased markedly (54%) even with only an oral 2-gram dose of ascorbate, while alone the reductant showed no increase (Conte et al., 1984). *Clearly, oral ascorbate given with a chelator can substantially mobilize and enhance clearance of iron from the body.* However, it may also, depending upon chelator and conditions, enhance ROS formation as found with incomplete DFP and distorted EDTA coordination, as explained earlier. Indeed, IV EDTA and ascorbate given together does somewhat increase markers for patient plasma ROS damage (Hininger et al., 2005), but this should be controllable with other chelators. *Also, important to note is that in vitro ascorbate has intrinsic biphasic behavior where at low concentrations it can generate ROS but at higher concentrations eliminates them* (Buettner and Jurkiewicz, 1996; Griffiths and Lunec, 2001), *which argues for treatment at higher concentrations.* Taken together, the clinical *in vivo* results agree with ascorbate-induced *in vitro* molecular level protein aggregation reversal highlighting enhanced mobilization and elimination of iron as it becomes reduced.

## Potential parameters of treatment

The simultaneous use of a chelator with a reductant such as ascorbate or glutathione may prove useful in mobilization and removal of IBI pathologically associated with neurodegeneration. The dosage and sequencing are yet to be determined, but this could easily be approached with animal models. Parameters determining the efficacy would be identity of the chelator-reductant combination and their plasma concentration clearance profiles. However other parameters such as treatment sequence, length, and administration on-off cycle may be very important. More advance treatments may include administration of reductant precursors, multiple chelators, and multiple reductants. Ascorbate and reduced glutathione levels are closely intertwined (Crichton, 2009). Potential difficulties could be ROS generation by a bidentate chelator if its concentration is too low or lack of reductant ROS scavenging if reductant is too low. Again, ascorbate is an effective scavenger of ROS if its concentration is sufficiently large. The short (reduced) ascorbate half-life may be an advantage with initial high reductant concentration reducing and freeing iron from the protein aggregates and then oxidizing iron becoming tightly bound to chelators for elimination. Potential problems should be controllable by optimization of the eight parameters above. Worth repeating is that neurodegeneration is not a classic iron overload condition, and the iron removal would likely need to be more subtly accomplished, perhaps over several treatments or long-term. Finally, there is a clinical warning not to use the chelator DFO together with ascorbate for iron overload therapy concerning cardiac risk, but this may not be applicable with the comparatively low iron levels in neurodegenerative diseases versus those with systemic iron overload.

## Summary

It is my perspective that there is enough deductive and experimental evidence to comprehensively investigate the combined use of chelator with reductant as treatment for neurodegenerative diseases. This approach targets IBI-induced protein aggregation and ROS formation, and focusses on the hallmarks of aging cells: increased iron, decreased reductants, and decreased autophagy. There are sufficient appropriate neurodegenerative disease animal models to test, and in iron overload disease this combined iron chelator-reductant therapy has already proven effective in mobilizing and eliminating iron. Although oral administration of ascorbate with chelator may prove effective, IV administration of reductant may be necessary for a variety of physiological and chemical reasons, and it appears to be a non-toxic approach in general.

## Data availability statement

The original contributions presented in this study are included in the article/supplementary material, further inquiries can be directed to the corresponding author.

## Author contributions

The author confirms being the sole contributor of this work and has approved it for publication.
